# Characterisation of re-entrant circuit (or rotational activity) in vitro using the HL1-6 myocyte cell line

**DOI:** 10.1016/j.yjmcc.2018.05.002

**Published:** 2018-06

**Authors:** Charles Houston, Konstantinos N. Tzortzis, Caroline Roney, Andrea Saglietto, David S. Pitcher, Chris D. Cantwell, Rasheda A. Chowdhury, Fu Siong Ng, Nicholas S. Peters, Emmanuel Dupont

**Affiliations:** Myocardial Function, National Heart and Lung Institute, Imperial College London, London, UK

**Keywords:** Re-entry, Rotational activity, Triggers, HL1-6 myocytes, Monolayer culture, Optical mapping

## Abstract

Fibrillation is the most common arrhythmia observed in clinical practice. Understanding of the mechanisms underlying its initiation and maintenance remains incomplete. Functional re-entries are potential drivers of the arrhythmia. Two main concepts are still debated, the “leading circle” and the “spiral wave or rotor” theories. The homogeneous subclone of the HL1 atrial-derived cardiomyocyte cell line, HL1-6, spontaneously exhibits re-entry on a microscopic scale due to its slow conduction velocity and the presence of triggers, making it possible to examine re-entry at the cellular level.

We therefore investigated the re-entry cores in cell monolayers through the use of fluorescence optical mapping at high spatiotemporal resolution in order to obtain insights into the mechanisms of re-entry.

Re-entries in HL1-6 myocytes required at least two triggers and a minimum colony area to initiate (3.5 to 6.4 mm^2^). After electrical activity was completely stopped and re-started by varying the extracellular K^+^ concentration, re-entries never returned to the same location while 35% of triggers re-appeared at the same position. A conduction delay algorithm also allows visualisation of the core of the re-entries. This work has revealed that the core of re-entries is conduction blocks constituted by lines and/or groups of cells rather than the round area assumed by the other concepts of functional re-entry. This highlights the importance of experimentation at the microscopic level in the study of re-entry mechanisms.

## Highlights

• The cell line HL1-6 makes it possible to examine propagation at the single cell level.
• A new analysis toolkit permits visualisation of re-entry core in 2D cultures.
• The number of triggers and re-entries depends on colony size.
• Re-entry cores are groups of cells connected by thin lines of functional block.
• The mechanism of re-entry appears comparable with the leading circle concept.

## 1 Introduction

Fibrillation requires two components. Firstly, there must be abnormal activation activity, known as the trigger(s). Secondly, there must be a vulnerable substrate that enables fibrillation to perpetuate [1,2]. The triggering event is thought to arise from two possible sources: either directly from focal ectopic activity or from re-entry [3,4]. Throughout this study, re-entries (or rotational activities) are defined as regular wavefronts rotating around a central core.

Focal activity may originate from propagation of after-depolarisations from individual myocytes. However, although after-depolarisation has been observed in single cells, it would be unlikely to propagate in a well coupled tissue [5]. Some regions of the heart such as infarct border zones or the thin cardiac tissue at the pulmonary veins may allow this abnormal activity to overcome the effects of source-sink mismatch that would be experienced elsewhere in the myocardium [3,6]. Unfortunately, it is difficult to identify the specific triggering event, since micro re-entry and propagated after-depolarisations cannot be distinguished from each other at the macroscopic level of clinical studies [7].

Functional re-entry emerged as a concept in the 1970s with the *leading circle* hypothesis [8]. This hypothesis suggests that wavefronts rotate around a core of unexcitable cells. The core is unable to propagate action potentials as it is kept in a depolarised, constant refractory state by incoming centripetal wavefronts [[8], [9], [10]].

The other hypothesis of functional re-entry is the *spiral wave* theory, from which the term *rotor* appeared. In this concept, the wavefronts of the spiral waves have increasing convexity towards the core which results in increasing source-sink mismatch and are unable to provide enough depolarising current to excite the core known as the singularity point [10,11]. Since the cells constituting the singularity point are excitable, the rotor is able to drift [2,11,12].

Both hypothesis propose that fibrillation is driven by re-entries, which emit waves of electrical activity, regardless of their mechanisms [2,13].

The HL1-6 cell line, a subclone of the original HL-1 cells [14], is functionally more homogeneous than the original HL1 line [15]. They maintain their differentiation and can be passaged indefinitely in culture. HL1-6 cells possess the ion channels necessary for generating action potentials and express connexins 40, 43 and 45 for gap junctional electrical coupling. Like primary neonatal cardiomyocytes, they propagate electrical impulses, albeit with approximately an eight times slower conduction velocity (~41 mm/s in HL1-6 [15] compared to ~34 cm/s in primary myocytes [16]). As in the original HL-1 line, HL1-6 myocytes display triggers and re-entry.

The aim of this study was to characterise the cores of re-entry. The slow propagation of the HL1-6 clone [15] allows this aim to be investigated using the latest high-speed optical mapping and computational analysis techniques. Fluorescence imaging of cell morphology and activity provided the unique ability to study features at the core of re-entry at a spatiotemporal level (single cell) not previously possible.

Estimates of the required number of triggers and colony sizes for re-entry to develop were obtained. Furthermore, we assessed whether natural triggers and re-entrant circuits are permanent and/or functionally determined features and characterise the core of re-entrant circuits by comparing re-entry cores with cellular morphology and activity.

## 2 Materials and methods

### 2.1 Cell culture

All cell culture work was carried out in laminar flow safety cabinets to maintain sterile conditions. HL-1 subclone 6 (HL1-6) [15] were grown in Claycomb medium (Sigma-Aldrich, USA) supplemented with 10% fetal bovine serum (Gibco, USA), 100 U/ml:100 μg/ml Penicillin/Streptomycin (Sigma-Aldrich, USA), 2 mM l-Glutamine (Sigma-Aldrich, USA) and 0.1 mM Norepinephrine (Sigma-Aldrich, USA).

Cells were maintained in 100 mm diameter TC-treated culture dishes (Corning, USA) coated with a 5 μg/ml solution of fibronectin (Sigma-Aldrich, USA) in Hank's Balanced Salt Solution (HBSS) (Thermo Fisher Scientific, USA) for 30 min.

Cells were split in ratios from 1:6 to 1:3 once dishes reached confluency. Using 0.05% trypsin/EDTA (Sigma-Aldrich, USA) in HBSS and incubated in 1% CO_2_ at 37 °C for approximately 10 min. After dilution in Claycomb medium, the single cell suspension was re-seeded in new coated 100 mm dishes.

### 2.2 Seeding round colonies of controlled area

A range of volumes of fibronectin solution was applied to 35 mm uncoated low-walled μ-dishes (ibidi, Germany) to achieve cell colonies of consistent sizes. Drops of the solution formed circular shapes due to the hydrophobic nature of the dish surface. Drop volumes were set at 2.5, 5 and 10 μl. A large drop of 150 μl was also applied to facilitate checking for cellular activity.

After application, the fibronectin drops were left for approximately 30 min and then aspirated from the dish surface. 350 μl from a 2.6 ml single cell suspension from a confluent 100 mm dish was added directly to the μ-dishes and incubated in 5% CO_2_ at 37 °C for approximately 30 min. Extensive wash with HBSS removed excess cells not adhered to the surface (i.e. outside the drop of fibronectin coated areas). Growth medium was added and dishes returned to the incubator. Cells in these colonies would typically exhibit intrinsic activity after four to eight days in culture.

### 2.3 Fluorescence microscopy imaging

Prior to all optical mapping experiments, the cell colonies were loaded with Fluo-4 AM diluted in HBSS containing 1 mM CaCl_2_ at a concentration of 10 ng/ml for approximately 20 min to visualise Ca^2+^ transients as a surrogate for action potentials and to visualise propagation and identify re-entry. Ca^2+^ transients are used as a surrogate due to the much larger fluorescence amplitude recorded compared to voltage dyes (e.g. di-8-ANEPPS). This makes it possible to visually track wavefronts to locate the re-entry cores within monolayers, which is impossible with voltage dyes. Cells were returned to the incubator for 20 min to allow the dye to de-esterify before imaging was carried out. Occasionally, cells were also stained with 40 μM di-8-ANNEPS (Molecular Probes, Invitrogen) diluted in 1 ml HBSS supplemented with 0.05% Pluronic F-127 (Life Technologies, USA) for 2 min. Any longer time resulted in complete cessation of activities seen using Fluo-4.

An Axio Observer microscope (Zeiss, Germany) was used to record Ca^2+^ transients of the colonies. Cells were subjected to light at a wavelength of 494 nm and light was then captured from the emission spectrum at 516 nm. Video recordings using a high aperture 10× lens were taken at 100 frames per second using an exposure time of 2 ms and 4×4 spatial binning (2.6 μm/pixel). Images with no spatial binning (0.65 μm/pixel) were captured immediately following recordings. These images were used to align data from following experiments to the original video recording. All recordings on this system were captured using ZEN Pro imaging software (Zeiss, Germany) and stored in the proprietary CZI format.

Dual voltage- and Ca^2+^ transient optical mapping was captured simultaneously using a custom-built microscope setup (Cairn Research, UK). The system was built around an upright microscope (Eclipse FN1, Nikon Instruments Europe B.V.). Excitation light (470 nm) was supplied by an OptoLED system (Cairn Research, UK). The emitted fluorescence was collected by a high aperture 20× water dipping lens (Olympus). Using a 560 nm edge BrightLine single-edge dichroic beamsplitter, located in an Optosplit II “LS” emission image splitter (Cairn Research, UK), the fluorescent light was divided into two beams that were passed through emission filters (525 nm and 628 nm). Recordings were captured at 100 frames per second with 4 × 4 spatial binning (1.15 μm/pixel) and 1 ms exposure time by a Zyla 10-tap sCMOS camera (Andor Technology, UK).

### 2.4 Imaging cell morphology

For experiments using the Zeiss Axio Observer microscope, cell morphology was captured by live staining the cell membranes with 1 mg/ml Wheat Germ Agglutinin (Sigma-Aldrich, USA) diluted 1/40 in HBSS containing 1% bovine serum albumin for 10 min then rinsed thoroughly with HBSS before imaging. Live staining ensured that morphology was not affected by fixation of the cell.

*Z*-stack images of cell morphology were captured using the microscopy setup for μ-dishes described previously. Images were captured with a z-axis resolution of 0.5 μm. The z-stack images were subsequently deconvolved using Huygens software (Scientific Volume Imagine, The Netherlands).

### 2.5 Quantification of natural triggers to cell colony size

In preliminary experiments, attempts were made to visually identify individual triggers in each size of colony. This process was challenging and unfeasible in limited time with the microscope system. Instead, 30 second videos were recorded at the centre of each colony. Natural triggers are characterised by wavefronts of different angle, direction, low and irregular frequency and were used as a surrogate for activity from individual natural triggers.

Each recording was additionally classified for the occurrence of re-entry in the colony. *Re*-entry was identified by over four very regular consecutive wavefronts with the same characteristics (e.g. direction, frequency). When re-entry was observed, the wavefront counts were excluded from the subsequent analysis as trigger activities would be impeded by wavefronts originating from the re-entry(ies).

### 2.6 Spatial stability of natural triggers and re-entrant circuits

Medium was aspirated and replaced with HBSS containing 20 mM KCl to depolarise the cell membranes and maintain the fast Na^+^ channels in the inactivated state or containing 3 mM Heptanol (stock 1 M in isopropanol) to interrupt gap junctional coupling. Each colony was checked to ensure activity had ceased within a few seconds. The KCl/HBSS or heptanol/HBSS solution was removed, the cells rinsed and returned to HBSS containing 1 mM CaCl_2_. Further 10 second recordings were taken at each location where triggers or re-entries were previously observed.

### 2.7 Fluorescence microscopy data processing and analysis

All images and videos were analysed using FIJI open source software (version 1.51f, https://fiji.sc/). Custom optical mapping tools written in MATLAB (version 2016a, Mathworks, USA) were extended from previous work in the lab group [17,18]. The development and optimisation of this code was necessary to analyse the data obtained during this study. The validation and description of this code are described in detail in the Supplementary information. In assigning activation times to pixels in optical mapping, we adopt the standard convention and define activation time as the point of steepest upstroke in the fluorescence signal [17].

### 2.8 Statistical analysis

All values are presented as the mean ± standard error to the mean. n represents the number of times an experiment was repeated. Normality was tested using the Kolmogorov-Smirnoff test. Differences between proportions were tested using Fisher's exact test followed by a Bonferroni correction for multiple comparisons. Mann-Whitney *U* test was used to test for significant differences between groups and corrected for multiple comparisons using Dunn's test. Relationships between two measurement variables were evaluated with linear regression. Differences were judged significant when *p* < .05. All statistical analyses were completed using R open source software (version 3.3.2, available from https://www.r-project.org).

## 3 Results

### 3.1 Relationship between colony area and occurrence of natural trigger cells

The resulting colony areas for different volumes of fibronectin drops were established as 2.5 μl: (3.5 ± 0.1) mm^2^ (*n* = 5), 5 μl: (6.4 ± 0.1) mm^2^ (*n* = 3) and 10 μl: (9.8 ± 0.2) mm^2^ (*n* = 3) from the phase images analysed. These values are adopted as the areas of colonies in the analyses that follow.

**Fig. 1:**
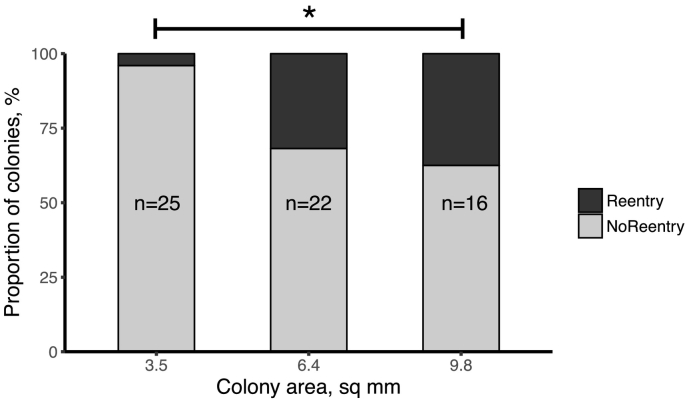
Proportion of colonies with observed re-entry wavefronts for different colony areas. Number of recordings for each area are indicated. *p < .05.

A total of 63 recordings were captured and analysed in various sizes of colonies (3.5 mm^2^, *n* = 25, 6.4mm^2^: *n* = 22, 9.8mm^2^: *n* = 16). Re-entry (or a series of wavefronts originating from re-entry) was observed most often in 9.8mm^2^ colonies (38%, *n* = 6/16), a smaller proportion in the 6.4 mm^2^ size (32%, *n* = 7/22) and only one identified in the smallest 3.5mm^2^ colonies (4%, *n* = 1/25). Fig. 1 summarises the occurrence of re-entry with colony area. There was a significant increase of re-entry occurrence from the smallest to largest colony sizes (*p* = .028, Fisher's exact test with Bonferroni correction) but no significant difference between the small and medium colonies or the medium and large colonies (both *p* > .05).

**Fig. 2:**
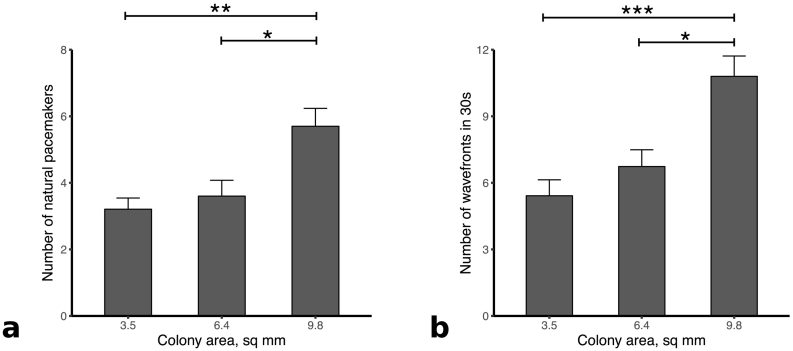
(a) Number of natural triggers at each colony size for HL1-6, with significance levels indicated (b) Total number of wavefronts in 30 second recording at each colony size. Significance levels using the Mann-Whitney *U* test with Dunn's correction are indicated. Error bars are standard error to the mean. *p < .05, **p < .01.

Wavefronts from re-entry in the colonies would affect the count for triggers in the subsequent analysis. After excluding recordings with re-entry, the number of natural triggers was quantified for each colony size. The results were 3.5 mm^2^: 3.2 ± 0.3 triggers (*n* = 24), 6.4 mm^2^: 3.6 ± 0.5 triggers (*n* = 15), and 9.8mm^2^: 5.7 ± 0.5 triggers (*n* = 10). As expected, there was a significant increase in number of both triggers and wavefronts between the 3.5 mm^2^ and 9.8 mm^2^ colonies (*p* = .0014) and between the 6.4 mm^2^ and 9.8 mm^2^ colonies (*p* = .0166). Fig. 2a displays these results.

To determine the change in general activity with colony area, the total number of wavefronts regardless of direction or frequency was also quantified during each 30 second recording. In these experiments, a significant difference in wavefront activity was observed between the 3.5 mm^2^ and 9.8 mm^2^ colonies (*p* = .003) and between the 6.4 mm^2^ and 9.8 mm^2^ colonies (*p* = .0264). Fig. 2b summarises these data.

### 3.2 Spatial stability of natural triggers and re-entrant circuits

**Fig. 3:**
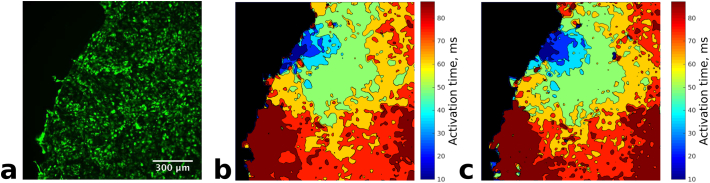
Activation map of a natural trigger at the edge of a colony. (a) fluo4-AM stained cells, (b) activation map and (c) activation map after depolarization with KCl and reactivation in normal medium. Both maps are virtually identical.

20 natural triggers in HL1-6 were captured before and after depolarisation with high K^+^ extracellular solution and 13 before and after inhibition of gap junctional coupling by heptanol. Natural triggers were generally characterised by automatic activity at irregular frequencies. After confirmation of the location of the trigger cells in the pre-treatments recording, activity was evaluated (Fig. 3). Analysis of the recordings showed that after depolarisation 10% (*n* = 2/20) of recordings exhibited no activity, 55% (*n* = 11/20) showed activity from another location and 35% (*n* = 7/20) resulted in return of activity from the original trigger. After uncoupling by heptanol 38% (*n* = 5/13) showed no activity, 23% (*n* = 3/13) showed irregular activity from another location and 38% (n = 5/13) showed activity from the original trigger. No re-entry was detected after either depolarisation or uncoupling.

9 re-entrant circuits that occurred naturally in the colonies were recorded before and after depolarisation and 18 before and after electrical uncoupling to assess whether they returned to the same location. After both depolarisation and uncoupling, none of the re-entries returned to the same location in the data analysed. In 56% (*n* = 5/9) of the recordings after depolarisation and 28% (*n* = 5/18) after uncoupling, regular activity was observed from another location, suggesting a re-entry had developed elsewhere in the cell colony. 33% (*n* = 3/9) of recordings after depolarisation and 11% (*n* = 2/18) after uncoupling were affected by irregular waves from multiple directions, suggesting only natural trigger activity. 11% (*n* = 1/9) after depolarisation and 61% (*n* = 11/18) after uncoupling showed no activity. When activity was present after depolarisation or uncoupling, the original core locations displayed normal propagation of wavefronts indicating that the cells constituting the core were not dysfunctional.

### 3.3 Re-entry characteristics

In this study, we define conduction block as a very large delay which was observed at the core of the re-entries. The conduction block may therefore be composed of cells that can be activated but do not transmit these activities. Of the 27 re-entrant circuits captured over the course of this study re-entry was observed to remain stable around a fixed core for the duration of recordings (10 s) and the time to track them visually (up to several minutes). The mean dominant frequency was 2.7 ± 1.0 Hz.

The cores of re-entry were visualised over the full time of each recording (10 s) using our conduction delay detection algorithm. This algorithm highlights points in the recording that are in close proximity but have large difference in activation time, as a surrogate for conduction block (further details in Supplementary information).

**Fig. 4:**
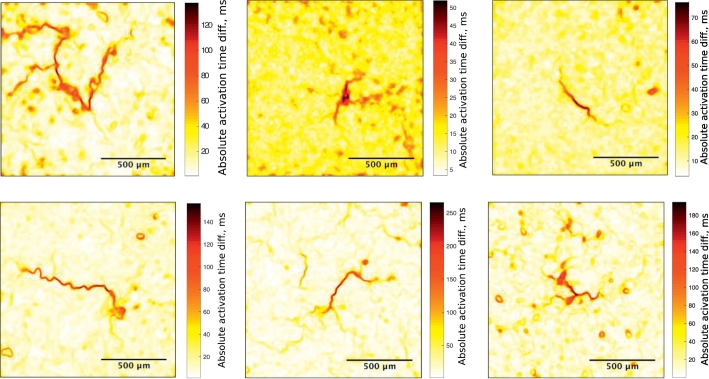
Examples of re-entry core shapes and sizes. Dark areas correspond to large conduction delay between each side of the area. Scale bars are different between images because the frequency of re-entry was different.

Re-entry exhibited a wide range of core characteristics (Fig. 4). 59% (*n* = 16/27) of re-entries were rotating around small areas of cells connected by thin lines of conduction block. In the other re-entries, 19% (*n* = 5/27) appeared to be rotating around a single line of conduction block and 22% (*n* = 6/27) around three or more connected lines of block.

**Fig. 5:**
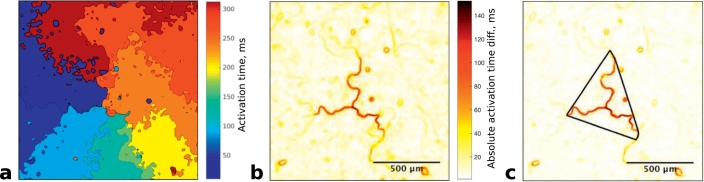
(a) Activation map of re-entry circuit in HL1-6, dominant frequency = 2.9 ± 0.1 Hz (b) Visualisation of re-entry core from **a** composed of multiple connected lines (c) Visualisation of re-entry core from **a** with calculated perimeter marked.

The perimeter around cores was estimated for each re-entry by fitting a convex hull to a thresholded image of the core and calculating its perimeter. A convex hull is mathematically defined as the smallest convex shape that contains a set of points and is a method to determine the shortest path around the core. Fig. 5 gives an example of a re-entry core with corresponding activation time map and core perimeter, the areas of conduction block can also be seen on the activation map.

**Fig. 6:**
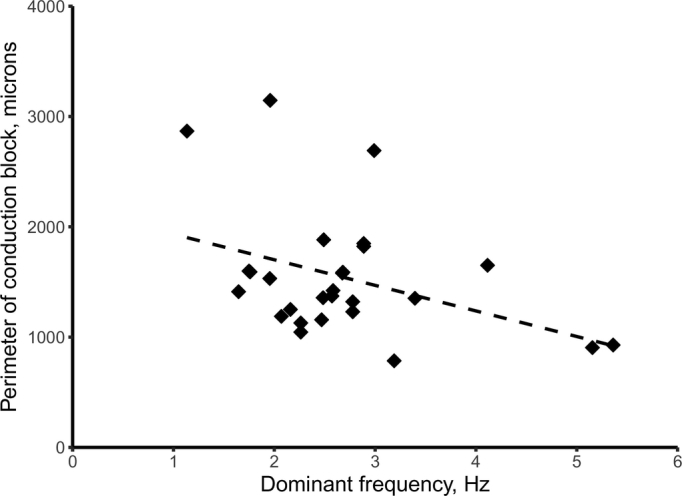
Scatter plot showing perimeter of re-entry circuit core vs dominant frequency of re-entry. Linear regression line, *p* = .044, R^2^ = 0.15.

A wide range of perimeters was observed between approximately 0.8 mm and 3.1 mm. The mean perimeter was 1.5 ± 0.6 mm. The perimeters of the re-entry cores were compared to their dominant frequencies. These results suggest that an increasing dominant frequency is associated with a smaller re-entry perimeter, however additional variables may also impact this relationship as indicated by the relatively low R^2^ value of the linear regression fit (Fig. 6).

### 3.4 Cell morphology and activity at re-entry cores

**Fig. 7:**
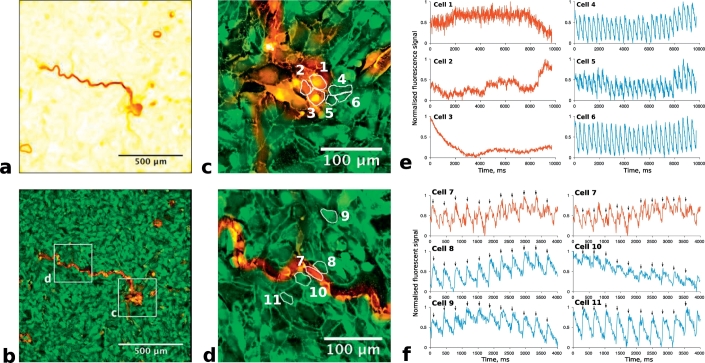
(a) Conduction block visualisation of re-entry in HL1-6 with dominant frequency of 2.9 ± 0.1 Hz. (b) Conduction block overlay on Wheat Germ Agglutinin live staining of cell membranes with areas of interest marked. (c) Zoomed image of conduction block area from (b) area c. (d) Zoomed image of conduction block line from (b) area d. Cells for further analysis are marked on zoomed image c and d. (e) Unfiltered calcium fluctuations for cells indicated in micrograph c. (f) Unfiltered calcium fluctuations for cells indicated in micrograph d.

The morphology and activity of cells at the core of re-entrant circuits was analysed by overlaying the conduction block heatmap on images of cell morphology from Wheat Germ Agglutinin staining. Fig. 7a shows the generated heatmap from a re-entry circuit composed of a single line connected to a small area of cells. In Fig. 7b, this heatmap is overlaid on a fluorescence image showing cell morphology.

By overlaying the conduction delay heatmap, we found that the areas of delay (or block) were not between cells but were found to be composed of small groups or lines of cells. Fig. 7c and d shows a zoomed view of these regions. Ca^2+^ transient activity during the recording was measured on an individual cell basis in the cells highlighted in Fig. 7c and d, and is shown in Fig. 7e and f with cell number indicated. Three cells are within the area of block (cells 1, 2 and 3) and three are outside and adjacent (cells 4, 5 and 6). As can be seen in Fig. 7e, cells 1, 2 and 3 display random fluctuations of intracellular Ca^2+^ while cells immediately adjacent (cells 4, 5 and 6) show very regular Ca^2+^ transients in synchrony with the re-entry.

The width of conduction block lines was generally found to be one or two-cell thick. Fig. 7d presents a close view of this feature. Ca^2+^ activity was measured in cells highlighted in Fig. 7d. These were chosen such that they include a cell within the line of block (cell 7), a neighbouring cell on either side (cells 8 and 10) and cells further from the line which should exhibit normal activity with the re-entry circuit (cells 9 and 11). Fig. 7f shows the Ca^2+^ activity in these cells for a period of 4 s during the recording. The graphs in the top row both show the same activity from cell 7 to aid vertical comparison with other plots. The left column of Fig. 7f shows activity in cells above the line of block (cells 8 and 9) and the right column gives activity in cells below the line of block (cells 10 and 11). Arrows mark the periodic Ca^2+^ transients for each side. Signals are normalised but unfiltered to avoid any alteration to the raw data.

The activity recorded in cells far from the line of block (cells 9 and 11, bottom row) exhibits the expected periodic Ca^2+^ transients associated with the re-entry circuit with a dominant frequency of 2.9 Hz. Cells 9 and 11 are out of phase with each other as they are on opposite sides of the line.

The cell in the line of block (cell 7, top row of plot) exhibits different activity compared to the other cells. It appears to be firing Ca^2+^ transient at double the rotational rate of the re-entry circuit. The arrows in both plots indicate how this firing rate is consistent with the activity on either side of the conduction block.

Analysis of other cells in the line of block showed either similar activity to cell 7, or random fluctuations like cells in the area of block. To ensure that the observed firing rate of cell 7 was not due to the area recorded for the cell overlapping into adjacent cells, the analysis was repeated for smaller regions of cell 7 centre. The same activity was observed but with increased noise (as fewer pixels were being averaged in these cases).

**Fig. 8:**
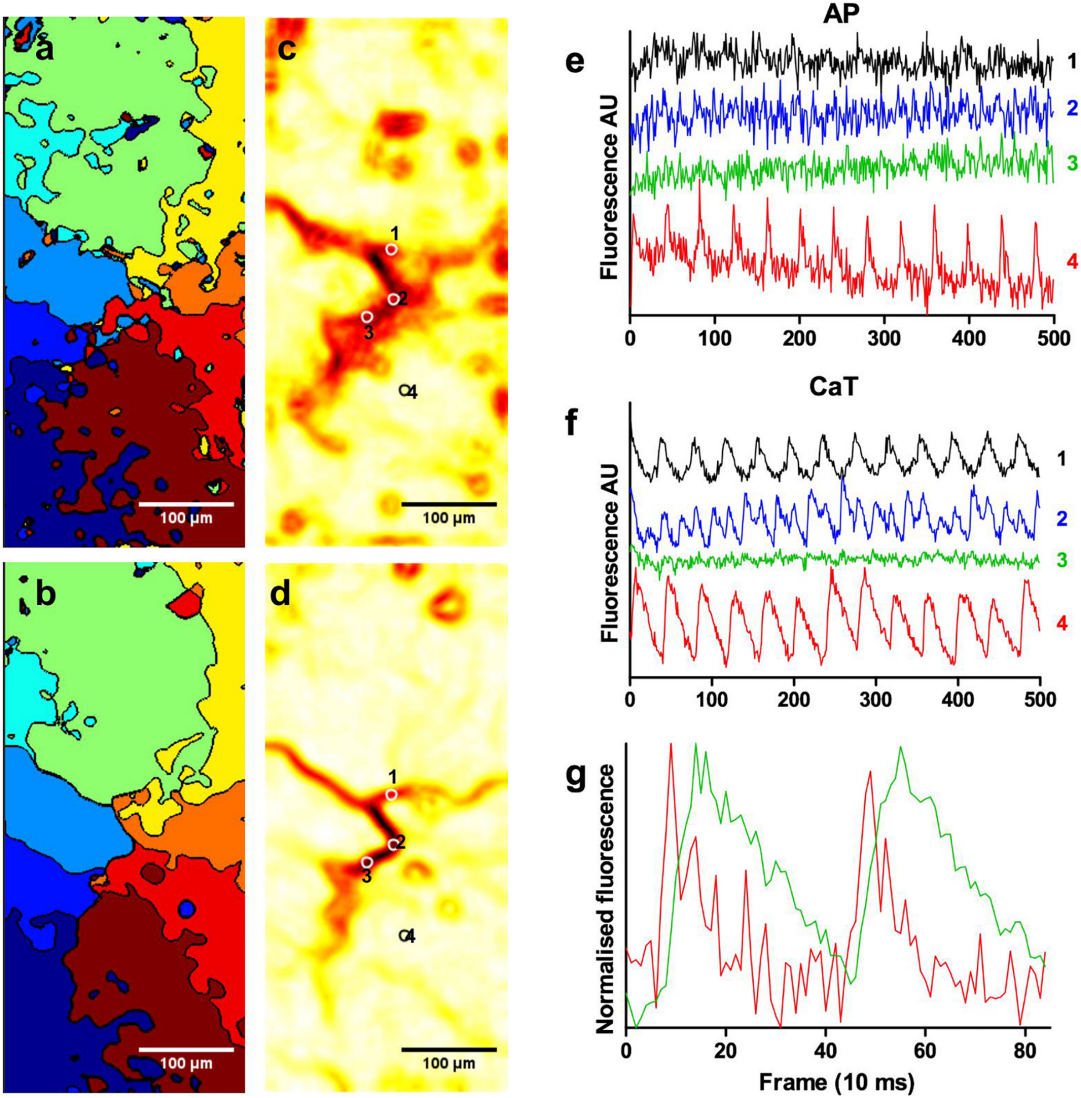
Simultaneous optical recording of action potential and Ca^2+^ transients. (a) and (b) show activation maps obtained for action potentials and Ca^2+^ transients respectively. The maps using Ca^2+^ transients are determined from data three frames later because of the delay between action potentials and Ca^2+^ transient upstrokes (see graph (g)). (c) and (d) are the corresponding conduction block areas. The areas further analysed are indicated by circles of 10 μm diameter. (e) and (f) show the traces for action potentials (AP) and Ca^2+^ transients (CaT) respectively. The number on the right of each trace correspond to the areas shown in (c) and (d). All traces in (e) and (f) are unfiltered and at the same scale (no normalisation). (g) shows normalised traces of two action potentials (red) and Ca^2+^ transients (green) of area 4 in (c) and (d) showing the delay between the upstrokes. (For interpretation of the references to colour in this figure legend, the reader is referred to the web version of this article.)

Despite the toxicity of di-8-ANEPPS to HL1-6 cells, we were able to carry out 3 simultaneous, dual optical mapping experiments to confirm that propagation patterns observed in Ca^2+^ transients are consistent with action potential propagation (Fig. 8).

Fig. 8a and b show that the activation map generated from action potentials and Ca^2+^ transients are similar. The maps were calculated using the same recording with a constant frame shift of three frames to account for the delay in upstroke of the Ca^2+^ transient compared to action potential (Fig. 8g). Fig. 8c and d show the conduction block maps for voltage and Ca^2+^ transients respectively. The conduction block maps show a similar core morphology but the core generated from action potential analysis is larger than with Ca^2+^ transients. This may be due to the much higher noise and lower signals produced by di-8-ANEPPS compared to Fluo-4.

Fig. 8e and f show the temporal signals taken from the indicated areas around the rotation. Using this instrument, we could not obtain the cellular context because the set up used does not allow us to obtain Z stacks for deconvolution. Regardless, the indicated areas (10 μm diameter) are smaller than a single cell. Outside of the core (trace 4), both fluorescent signals clearly show firing patterns with a corresponding frequency. In areas at the centre of the rotation (traces 1, 2 and 3), no action potentials were detected although some rhythmic activity slightly above the high noise can be seen in trace 1 of Fig. 8e that could indicate a low amplitude Ca^2+^ dependant action potential. This was a general observation of areas tested inside the core of the map generated from the voltage sensitive probe. However Ca^2+^ transients can be seen at the frequency of re-entry in area 1, at twice the frequency in area 2 and are not present in area 3. This likely indicates that the cells in the core are depolarised above the threshold for the Na^+^ channel activation but not always above the threshold for Ca^2+^ channel activation and are therefore capable of releasing Ca^2+^ from the sarcoplasmic reticulum as Ca^2+^ transients triggered by Ca^2+^ influx through Ca^2+^ channels (Ca^2+^ dependant action potential). This observation is similar to the analysis in Fig. 7 where the core cells may display no Ca^2+^ transient (cells 1, 2 and 3 in Fig. 7) or at twice the frequency of re-entry (cell 7 in Fig. 7).

## 4 Discussion

The aim of this study was to obtain insight into the formation and the mechanisms of re-entry (or rotational activity). We found that 1) the number of triggers and re-entrant circuits depends on colony size, 2) the triggers appear more stable while re-entries never reappeared at the same location, 3) the core of the re-entries is not only a simple round small mass of cells but also connected lines(s) of conduction block, 4) the periodicity of the re-entry depends on the size of the core (as estimated using the convex hull approach) and 5) the cells constituting the core are either inactive or are able to display Ca^2+^ transients but no detectable action potentials.

### 4.1 Re-entry can be initiated from few triggers but require a minimum area

The aim of the trigger quantification experiments was to determine if a minimum threshold for number of triggers or colony area exists for re-entry formation in HL1-6 monolayers. The results showed that there were significantly more trigger cells and wave activity in the largest colony (9.8 mm^2^) compared to the smaller sizes (3.5 mm^2^ and 6.4 mm^2^). However, an unavoidable limitation of this analysis is that the presence of re-entries may have skewed the number of triggers since no trigger can be detected in the presence of re-entry. Also, fast and regular trigger, a rare occurrence, could give the appearance of a re-entry with our method. Nevertheless, we found, as expected, that the number of automatic focal activities increased with colony size.

Despite the low incidence of natural triggers, it was possible to observe and record a re-entrant circuit in the smallest colony area. This suggests that only a small number of point sources are required for re-entry to develop. Previous studies have shown that it is possible to induce re-entry in cell monolayers using stimulation by S1S2 protocol or rapid pacing from a point source [2,19,20]. In our study, the smallest colonies had over three triggers on average yet showed almost no occurrence of re-entry. The significant increase of re-entry occurrence between the smallest and largest colonies indicates that sufficient area is a likely condition for re-entry to develop, provided that triggers are present. This is in agreement with the critical mass hypothesis whereby if the area of excitable tissue is too small and conduction too fast then re-entry cannot occur [21]. In HL1-6 monolayers, as colony size increases it appears that the ‘number of triggers’ condition is satisfied before the minimum area, but this may not be the case for other cell types with differing degrees of natural automaticity and/or conduction velocities. Given that the smallest HL1-6 colony size showed almost no re-entry while the medium size exhibited re-entrant activity in 32% of recordings, it can be inferred that the minimum area threshold in monolayers of this HL-1 subclone is between 3.5mm^2^ and 6.4mm^2^.

### 4.2 Natural triggers remain spatially stable while re-entrant circuits can form in multiple locations

After KCl depolarisation or electrical uncoupling by heptanol and reactivation in normal HBSS, triggers from cells that exhibited automaticity returned in the same location in 35% of cases using depolarisation and 38% after uncoupling. However, in the other recordings, activity from other triggers was observed to return at other location(s). This observation is potentially caused by changing frequency of other triggers in the colony impeding the original triggering cells. Similarly, these other triggers would likely have been “silenced” by the original trigger before depolarisation. Indeed, it would be expected for automaticity to remain a constant property of the cell(s) as it is a molecular mechanism that should not be affected by depolarisation of the cell membrane [22,23] or by uncoupling.

Re-entrant circuits initiated naturally were stable but never returned to the original location after either treatment. In a large number of cases, regular wavefronts were seen emanating from outside of the recorded area. These were likely to propagate from re-entry which had developed at another location and became stable.

These results would imply that many different sites exist as substrate that are conducive to re-entry formation. Complex interaction of wavefronts from trigger activity appears to arrange into an organised circuit that can manifest itself at any one of these locations. The consequence of this finding to clinical practice is that there may not be a single specific site that can be ablated to prevent initiation of re-entrant activity, as highlighted in a recent review by Josephson [24].

There was, however, some differences between depolarisation using KCl and uncoupling using heptanol. After heptanol washout, generally less activity was observed; for the triggers analysis, 38% of the colonies displayed no activity after heptanol washout while only 10% were devoid of activity after repolarisation. Similarly, for the re-entries analysis, repolarisation lead to the re-creation of re-entry in other locations in 56% of cases but heptanol washout resulted in only 28% of cases. This appear to be the case for the number of colonies displaying triggers after treatments (repolarisation 33% Vs heptanol washout 15%) or losing activity completely (repolarisation 11% Vs heptanol washout 46%). This may be caused by heptanol having activity on other channels and receptors since its mode of action rely non-specifically on membrane fluidity [25,26] while a slight elevation of K^+^ will be compensated rapidly by the cells after returning in normal HBSS.

### 4.3 The core of re-entrant circuits was characterised by conduction block lines and/or small areas of cells

The very large delay observed at the core of the re-entries is defined as conduction block and is not present between cells but is composed of cells likely in refractory period (see section 4.4 below). The conduction delay algorithm enables the analysis of cell morphology underlying re-entrant circuits in culture (see Supplementary information). Thin lines of conduction block were a prominent feature of the re-entry cores, an unexpected outcome of the results that has not been reported previously.

The core maps were generated from propagation patterns observed from Ca^2+^ transient imaging. This was necessary to visually track the propagating waves. The voltage dye does not permit visualisation of re-entry due to its low signal amplitude and could not be used routinely because of its high toxicity to HL1-6 cells that stopped all activities in most cases. However, our results from few simultaneous dual optical mapping studies indicate that the Ca^2+^ transients are a reasonable surrogate for action potentials under the reported conditions of this work.

Hong et al. [27] were the first to map a re-entry circuit in the original HL-1 cell line. They reported that re-entry observed in cell monolayers was very stable. Similarly, Umapathy et al. [28] showed that 32 of their 45 recordings of HL-1 cellular monolayers exhibited stable re-entrant circuits, with the remainder displaying only wave break and collision. These findings agree well with the characteristics of re-entrant circuits in the current work which all exhibited stable rotation around a fixed core.

The frequency of re-entrant circuits observed in this work also agrees well with previous studies. Hong et al. [27] reported a decreasing trend of re-entry rotation frequency in HL-1 cells with time in culture. They observed re-entrant circuits with frequencies between approximately 1.4 Hz and 3.3 Hz (determined from Figure 1B in [27]). 22 out of 27 recordings of re-entrant circuits from this study fall within that range. The outlying values are potentially a result of differences between the original HL-1 cell line and the more homogeneous subclone 6 used in these experiments [15], recordings taken after less time in culture and/or our seeding method that give confluent colonies just after seeding.

There was a significant difference between the dominant frequencies of re-entrant circuits. Results also suggest a relationship between the perimeter of the re-entry core and dominant frequency of re-entry. Increasing perimeters were related to lower rotation rates. This is an intuitive result as, in a re-entry with a larger core perimeter, waves of excitation must travel a greater distance to complete a circuit which would give a longer re-entry time (assuming conduction velocity remains the same). The consequence of this finding is that rotation frequency of re-entry may be determined by microscopic features at the core. HL1-6 is monoclonal and therefore homogeneous in terms of cell electrophysiological properties but not in cell volume which affects the effective refractory period. Small cells in the core will have a longer effective refractory period if connected to large cells that depolarise them at each rotation. This difference in cell sizes and effective refractory period could be responsible for the difference in re-entry core sizes.

To the authors' knowledge, no previous work has attempted to determine the size of re-entry cores at the level of detail of this study. In neonatal rat cardiomyocytes, a study of re-entrant circuits at lower resolution [20] approximated the core as an ellipse with a perimeter length of around 1.7 mm (calculated from the values reported in the results of [20]). This is close to the mean values from this work (1.5 mm) despite the slower conduction velocity exhibited by HL1-6 subclone [15]. However, the re-entry core was not shown to be elliptical in shape necessitating the development of a separate interpretation of core perimeter. It is feasible that at a lower resolution, propagation around conduction block lines could have the appearance of an elliptical area of cells.

### 4.4 Cells within the core of the re-entry circuit may exhibit activation activity

Most of the cells inside the areas of conduction block show no activity despite neighbouring cells exhibiting calcium transients at the expected frequency of the re-entry. This suggests that cells in these regions may be held in a constant refractory state caused by depolarisation above the threshold of both the Na^+^ and Ca^2+^ channels and thus unable to propagate activity. The observation that some cells still fire Ca^2+^ transients, sometime at twice the frequency of the re-entry, but without detectable action potentials suggests that the cells are likely depolarised above the Na^+^ channels threshold but below the threshold of the Ca^2+^ channels. A Ca^2+^ action potential of low amplitude would be very difficult to detect using this particular voltage probe because of its high toxicity that prevented us to stain the cells sufficiently to obtain high signal/noise ratios. Other voltage sensitive probes are currently being tested for routine use. This therefore suggests that cells which are part of the core cannot transmit enough charges to their neighbours resulting in a conduction block but are functional.

The characteristics of the re-entrant circuits in this work appear to exhibit many characteristics of the leading circle concept. According to this hypothesis, the fixed core of re-entry is in the refractory state (and thus unexcitable), due to continuously impeding centripetal wavefronts [8,9]. This agrees with cell activity observed inside the area of block. Here, cells remain unexcited and the core is fixed for the duration of the recording. A common argument against the leading circle hypothesis is that meander and drift have been shown to be important characteristics of re-entry during fibrillation [29,30]. In leading circle theory the refractory core remains in a fixed location. An explanation for this discrepancy could rest in the fact that in whole heart imaging it is only possible to study electrical dynamics on the endo- and epicardial surfaces. The visualised movement of the core may be due to scroll wave dynamics around a fixed location within the three-dimensional myocardium [31,32].

In contrast, features of re-entry observed in HL-1 monolayers do not appear to follow spiral wave (rotor) theory. This theory predicts that the re-entry circuit rotates around an excitable but unexcited core which does not necessarily remain fixed in one location [2,11,12]. All the re-entrant circuits observed in HL-1 colonies remained stable for an extended period of time, similar to other studies [27,28].

Rotor cores from spiral wave theory are also assumed to contain an area of cells that are not activated due to the source-sink mismatch from the increasing curvature of the wavefront towards the centre of rotation [2,11,12]. However, our results do not show increased curvature of the wavefronts close to the cores (for example, compare activation map and conduction delay heatmap in Fig. 7, Fig. 8). In the results of the current study, cores were characterised by small groups of cells and/or thin lines of conduction block. This discrepancy suggests that the form of source-sink mismatch maintained by spiral wave theory is not a determining feature in HL1-6 monolayers. Instead, source-sink mismatch due to cell size/volume and/or gap junctional coupling could be a factor in the location of re-entry formation.

Some of the differences between interpretations of re-entry cores may be explained by the disparate scales at which they are studied. Clinically-based cardiac mapping of re-entry is carried out at much lower spatial resolution than possible in our cell culture model, and tends to focus on larger areas and macroscopic features [[33], [34], [35]]. Therefore, what may be viewed as spiral waves in clinical studies could potentially be driven by microscopic re-entry similar to those observed in this work.

### 4.5 Limitations

The use of Ca^2+^ transients as a surrogate for action potential was a necessary limitation of the study that allowed us to visually track propagating wavefronts during experiments. While simultaneous optical mapping of Ca^2+^ transients and voltage has been studied previously in an HL-1 2D monolayer, the authors used a higher magnification lens and much shorter timescale that would not allow us to analyse the features of rotational activity at the scale and detail of this work [36]. Our experiments with voltage dyes (which were limited by photo bleaching effects and the toxicity of the dye to the cells) suggest that propagation patterns of voltage and Ca^2+^ transients are similar in our experimental setting. However, further testing is required before this can be confirmed, and our conclusions are therefore drawn predominantly from Ca^2+^ activity. There may be differences between the action potential and Ca^2+^ transients as it is possible for cells to continue to fire Ca^2+^ transients while the membrane potential is above the activation threshold for Na^+^ channels. The observation that cores are consistently larger when detected with voltage sensitive probe may indicate that we may be under-estimating the thickness of the lines of conduction block/slowing. Regardless, our results showing simultaneous mapping of re-entry cores indicate that the main features of conduction block/slowing correlate well.

Our protocol and assumptions to differentiate triggers (low frequency, irregular wavefronts) from re-entry (high frequency, very regular wavefronts) may have biased some results since, although rare, some triggers can be very regular and fall within the frequency of re-entry.

The 2D monolayers in HL1-6 cell culture also represent a necessarily simplified model of the 3D features that exist in vivo. While the exact mechanisms of re-entry initiation and maintenance may differ, action potential propagation through gap junctions occurs in this cell system in exactly the same manner as more complex models. The finding that a stable re-entrant circuit can exist in a homogeneous monolayer is important in revealing that additional variables such as tissue anisotropy or macroscopic source-sink mismatch are not necessary for rotational activity characteristic of arrhythmias to occur. However, the fact that these variables appear to not be necessary does not imply that they are not an important consideration. It is also possible that the rotational activity observed in this simplified experimental preparation is generated by different mechanisms than in cardiac tissue, where effects including heterogeneity in the structure (e.g. fibre orientation, fibrotic tissue) and function (e.g. changing cell phenotypes across the atria) could play a key role not investigated in this study.

The rectangular morphology of atrial/ventricular myocytes differs from the round shape of HL1-6 cells. In HL1-6, gap junctions are also distributed evenly around the outer membrane [15]. The different morphology and alignment of cells in myocardial tissue introduces a degree of anisotropy not present in our homogeneous model. Anisotropy leads to differences between conduction velocities longitudinal and transverse to the alignment direction and orientation discontinuities which can initiate and maintain arrhythmogenic behaviour [37]. We believe it is an important finding that microscopic rotational activity can exist in the absence of macroscopic anisotropy. However, future work could investigate this feature using micro-abrasion techniques [38].

Finally, HL1-6 cells display very low conduction velocities due to lower Na^+^ current and lower junctional conductance than atrial myocytes and this may favour rotational activity around a depolarised core. HL1-6 show similar protein expression patterns for connexins compared to mouse atria, but have approximately eight times lower junctional coupling compared to ventricular myocytes as assessed by the double voltage clamp technique on cell pairs (11.2 ± 3.1 nS vs 74 ± 17 nS) [15,39]. Patch clamp experiments show that the peak Na^+^ current is also about four times less (−85.1 pA/pF vs −324.7 pA/pF) [15,40]. Cells with larger Na^+^ current and higher junctional coupling, therefore faster propagation than our cell line, may produce spiral waves and rotors.

### 4.6 Conclusions

An expected result is that re-entry/rotational activity required sufficient triggers and colony area in which to form a stable circuit. However, our visualisation at single-cell resolution of the core of re-entry produced an unexpected combination of groups of cells and/or thin lines of conduction block rather than a round area of depolarised cells assumed by popular theories. Their location potentially depends on microscopic forms of source-sink mismatch and/or wavefront collisions that would produce small areas of cells in refractory state and therefore areas conducive to re-entry formation. Alternatively, there could be differences in connexins and/or Nav1.5 expression in the cells constituting the cores even though HL1-6 is monoclonal. We are currently examining this hypothesis by immunohistochemistry.

Our observations suggest that the core cells are depolarised and therefore in refractory period in a manner comparable with the leading circle hypothesis. The unexpected results obtained through this work such as the shape of the re-entry cores highlight the importance of further experimentation at the microscopic level. The fact that re-entries never come back to the same location but are very stable once formed may explain why atrial fibrillation becomes very stable with time.

## Appendix A Supplementary data

Supplementary material Image 1
