# Supplementary material for: Characterisation of re-entrant circuit (or rotational activity) in vitro using the HL1-6 myocyte cell line
Source: J Mol Cell Cardiol. 2018 Jun;119:155–64. doi: 10.1016/j.yjmcc.2018.05.002 (PMC6004038; doi:10.1016/j.yjmcc.2018.05.002)
Supplement: Supplementary file 1 — Supplementary material [file mmc1.docx]

**Supplementary Information:**

**Characterisation of re-entrant circuit (or rotational activity) *in vitro* using the HL1-6 myocyte cell line**

Charles Houston, Konstantinos N Tzortzis, Caroline Roney, Andrea Saglietto, David S Pitcher, Chris Cantwell, Rasheda A Chowdhury, Fu Siong Ng, Nicholas S Peters, Emmanuel Dupont.

Myocardial Function, National Heart and Lung Institute, Imperial College London, London, UK.

**Corresponding author:**

Emmanuel Dupont

Myocardial Function, National Heart and Lung Institute, Imperial College London, Imperial Centre for Translational and Experimental Medicine, 4th floor, Hammersmith Campus, Du Cane Road, London W12 0NN, UK.

Email: [e.dupont@imperial.ac.uk](mailto:charles.houston11@imperial.ac.uk)

Phone: +447522373612

**Development of fluorescence optical mapping processing and analysis tools**

The software tool developed has two main components: data processing and post-processing analysis. The processing steps follow the generally accepted best-practice workflow for optical mapping [1,2].

**Data processing**

*Data masking*

Recordings taken from the edges of small colonies include both light from the fluorescent dye in the cells and empty space around the colony; foreground and background pixels. Figure 1a gives an example. It is desirable to remove the background data before processing as it can skew subsequent results [1]. This was implemented by generating a *mask image* for the recording as follows.

First, the full video recording is averaged across all frames to generate a single mean image. The Canny edge detection algorithm [3] is applied to the averaged image. The algorithm only returns edges from within the region we aim to preserve (i.e. within the colony). These edges are dilated, which connects them together, and then holes in the resulting shape are filled. The largest fully connected area from this output is identified and all others discarded. This chosen area is again dilated and holes filled to give the final mask [1].

Figure 1b shows the result of applying this process to the microscopy image in Figure 1a. The algorithm picks out the relevant parts of the recording and dark background pixels are discarded.

**Fig. 1** (a) Fluorescence microscopy frame of recording taken from edge of colony. (b) Mask applied to image before processing steps

*Spatial filtering*

The purpose of spatial filtering is to reduce noise. Briefly, the process works by calculating an average for each pixel value with those of its neighbours within a defined area. The area used to determine neighbouring pixels is given by the size of the filter matrix or *kernel*. The kernel also defines any weighting applied to values from neighbouring pixels that are closer or further away.

The original code applied a square mean filter kernel for the spatial filtering step. This performed simple averaging to each pixel within a user-defined area. The process is repeated through each frame of a recording. The use of a mean kernel is common in tissue-scale optical mapping [1] but the heterogeneous waveforms seen on the monolayer-scale make it less appropriate for the current application. Thus, the spatial filtering step was altered to use a Gaussian filter kernel. This applies weighting to pixels further from the calculation point during convolution. The weighting values are determined by a Gaussian surface which can be defined by a single parameter,$\sigma$, for a square filter kernel.

Figure 2 gives a comparison between one frame of a re-entry circuit recording for the previous mean filter and the new Gaussian filter. Data was filtered at equivalent kernel sizes, a relationship defined as approximately $\sigma=(N-1)/4$ where $\sigma$ is the Gaussian parameter and $N$ is the size of the mean kernel. In Figure 2a, line artefacts are highlighted as a by-product of the mean filter kernel. Figure 2b shows that these artefacts do not occur when a Gaussian kernel is applied.

**Fig. 2** (a) False-colour frame of recording filtered using a mean kernel of $N=11$ pixels with areas of line artefacts highlighted. (b) The same false-colour frame of recording filtered using a Gaussian kernel of $\sigma=2.5$ with same areas highlighted as in a

A suitable value for $\sigma$ is selected by determining a balance between noise reduction and distortion of the underlying signal. Figure 3 shows the effect of increasing $\sigma$ on spatial filtering in a recording of natural trigger activity. In Figure 3a, the raw output signal with no filtering is affected by significant noise. Figure 3b shows that a low $\sigma$ value causes some of the underlying structure to emerge but noise remains. Lastly, Figure 3c displays how a more appropriate value of $\sigma=2.5$ reduces noise without having an impact on the useful signal. Further increases may lead to inappropriate spatial broadening of the data [1]. In the final version of the toolkit, the value of $\sigma$ is chosen based on the quality of the fluorescence recording. Generally, the value was held around 2.5.

**Fig. 3** Effect of different values of Gaussian parameter, $\sigma$, on the output of the spatial filtering process. (a) The raw fluorescence signal with no filtering applied. (b) The fluorescence signal after Gaussian filtering with $\sigma=0.5$. (c) The fluorescence signal after Gaussian filtering with $\sigma=2.5$

*Temporal filtering*

Temporal filtering is a further processing step used to remove noise that cannot be reduced by spatial filtering without distorting the underlying signal. The step works by first passing the discrete time signal through a fast Fourier transform. This converts the signal into a sum of weighted sinusoidal components of different frequencies which can be analysed in the frequency, as opposed to temporal, domain [1,4]. It is then possible to attenuate undesirable frequencies outside the band of interest through application of a digital filter. Different classes of filters exist, but Finite Impulse Response filters are generally applied to optical mapping data as they are inherently stable and only require a simple phase shift correction.

The original toolkit applied a Finite Impulse Response filter to each pixel through all frames of a recording. The low-pass frequency is user-defined and a study was conducted to assess suitable values for use on optical mapping data of the slow conduction velocity in HL1-6 monolayers. The appropriate level should be chosen to avoid smoothing of the optical Ca^2+^ transient upstroke of the signals, which can affect quantitative analysis. The steep gradient of the upstroke means it is characterised as a high frequency part of the signal so could be inadvertently affected by this processing step.

Figure 4 shows the effect of different low-pass frequencies on the time-filtered fluorescence signal. The data is shown normalised from a single pixel in a recording of an action potential response. Figure 4a indicates the lowest frequency level of 5Hz smooths the upstroke of the signal. Higher values reduce some of the noise while not affecting the underlying data. Figure 4b shows the first derivative of the signal from 4a to compare the maximum gradients at the upstroke. This plot shows that detail is being lost in the upstroke region with a low-pass frequency of 5Hz while other settings appear approximately equal.

**Fig. 4** (a) Normalised fluorescence signal of the Ca^2+^ transient upstroke at a single pixel in a recording after passing through a temporal filter at different settings for the low-pass frequency. (b) The first derivative of the normalised fluorescence signal in A. (c) Parameter sweep of low-pass frequency on the maximum first derivative of the fluorescence signal at the action potential upstroke

A parameter sweep of the low-pass frequency was carried out to inform a decision for an appropriate level. The measured output was the maximum first derivative at the action potential upstroke of the signal. Figure 4c shows the results. At high filter frequencies, the output stays relatively stable down to approximately 10Hz. Below this level, the maximum upstroke is reduced by the filtering step. The results suggest an optimum value, which removes as much noise as possible without losing fidelity in this region, exists between 10 and 15Hz. Therefore, we used 15Hz.

*Drift removal*

Drift removal was added to the processing steps of the software. The purpose of this stage is to attenuate low frequency drift in the signal [1]. Briefly, the fluorescence signal over time from each pixel is fitted to a fourth-order polynomial, which is then subtracted from the signal to form a new baseline. Figure 5 presents a graphical summary of all processing stages.

**Fig. 5** (a) Normalised fluorescence signal from a pixel in an unfiltered recording of natural trigger activity in HL-1 cells. (b) Corresponding fluorescence signal after passing through a spatial filter using a Gaussian kernel with $\sigma=2.5$. (c) Corresponding fluorescence signal after passing through a temporal filter with low-pass frequency of 15Hz. d: Final filtered signal after drift removal

**Data post-processing analysis**

After processing, a common issue encountered when analysing the electrical activity from optical mapping data of fibrillation is that waves of excitation can appear random and disordered [5]. One method to overcome this is through spectral analysis of the data after fast Fourier transform as described above. A critical aspect of using the fast Fourier transform is the Nyquist criterion. It states that in order to fully capture the range of existing frequencies, the sampling rate must be at least double the largest frequency of the signal [5]. Spectral resolution of a fast Fourier transform can be calculated from the sampling rate divided by the total number of samples [4].

Spectral analysis in fibrillation studies can also be employed as a means of calculating the dominant frequency. The dominant frequency is the frequency with the highest peak relative to the entire power spectra. It can be used to search for regular trigger activity or re-entrant circuits in recordings and quantify their characteristics. Sanders et al have shown that areas of high dominant frequency in cardiac mapping correlate well with locations of high organisation, which have been associated with the drivers of fibrillation [6].

Re-entry Vulnerability Index is a recently developed measure of the natural tendency of cardiac tissue to allow re-entry [7-9]. It uses the difference between repolarisation and activation times at different points in the tissue to assess this vulnerability and identify target areas for treatment. The algorithm has been applied successfully to *in silico* studies to identify exit sites of re-entrant circuits in scar-related ventricular tachycardia and was shown to work at resolutions as low as 8mm [7].

*Activation time maps*

Activation time had been previously implemented in the software. Briefly, it assumes activation occurs at the time of the steepest positive gradient in the fluorescence signal, $(dF/dt)_{\max}$, within a windowed portion of the recording. The window is chosen to cover one rotation of a re-entry circuit or one wavefront passing completely over the recorded area. Isocontour plots can be used to visualise the propagation of activation waves with time.

There are areas of cells that may not fire Calcium transients within the windowed signal but would still be assigned an activation time based on the above method. This can lead to misinterpretation of the maps produced. To alleviate this, a further *activation check* algorithm was added to the existing code which incorporates aspects of the method for defining a wavefront given by Bayly et al [10]. Briefly, an additional condition is imposed on the signal at its activation time: the peak immediately following the point of maximum upstroke must exceed a user-defined threshold level, otherwise, no activation is assumed to occur.

*Dominant frequency and regularity index*

Dominant frequency maps were added to the post-processing toolset to extract regular features from fluorescence recordings. Additionally, they can be used to determine the appropriate time window over which to calculate an activation map for one complete rotation.

The data is converted into the frequency domain via the fast Fourier Transform. The sampling rate of recordings was 100 frames per second so power spectra are obtained between 0 and 50Hz. The dominant frequency in each pixel can be identified by the highest peak on the power spectrum. The spectral resolution of the calculated frequencies can be evaluated as 0.1Hz for the 10 second recordings (100 frames per second divided by 1000 samples). Before the Fourier transform, the signal is windowed using a Tukey window, which reduces the amplitude of 2.5% of the signal smoothly to zero at the beginning and end of the recording [11]. This process diminishes interfering effects of discontinuities at the extremes of the domain which would affect subsequent analysis.

Figure 6 presents the dominant frequency maps and corresponding analysis for a re-entry circuit recorded in cells before and after the recording has been filtered. Figure 6a shows how two dominant frequencies appear to exist in the raw video: 0.1Hz and 2.9Hz. Figure 6b indicates how the filtering process has attenuated the lower dominant frequency and produces a more homogeneous map, with a single clear dominant frequency at 2.9Hz. Areas in this map which do not conform to the main frequency correspond to cell regions which do not activate or have activity independent of the wavefront of propagation.

**Fig. 6** (a) Dominant frequency map of re-entry circuit raw recording prior to processing stage. (b) Dominant frequency map of re-entry circuit after processing stage. (c) Raw power spectra from Point 1 on dominant frequency map. (d) Filtered power spectra from Point 1 on dominant frequency map. (e) Raw power spectra from Point 2 on dominant frequency map. (f) Filtered power spectra from Point 2 on dominant frequency map. (g) Regularity index map for raw recording data. (h) Regularity index map for filtered recording data

This result is emphasised by analysis of power spectra at Points 1 and 2 in the dominant frequency maps and presented in Figures 6c-f. In power spectra, generally only the locations of peaks are of interest and so no labelling is applied to the power axis in these graphs. Comparing Figures 6c and 6d (Point 1) shows that it is possible to identify the dominant frequency at this area before signal processing had taken place. In contrast, the stark difference between the peaks identified in Figures 6e and 6f (Point 2) emphasises how the filtering process attenuates undesirable noise to allow the underlying signal to emerge.

Regularity index maps were implemented to augment the analysis of dominant frequencies. As mentioned previously, these approximately correspond to the proportion of the signal that can be described by the identified dominant frequency. Figures 6g and 6h present the regularity index maps for this data before and after processing. The low values observed in Figure 6g highlight the noise present at multiple frequencies in the raw signal. Figure 6h shows that the effect of filtering is a general increase in the regularity index from noise attenuation. Both dominant frequency and regularity index maps also reveal cells which do not activate at the rate of the re-entrant circuit.

*Conduction block visualisation*

Conduction block is visualised by a novel algorithm developed as part of this project. As far as the author is aware, this is the first time this form of the algorithm has been applied to cell monolayer optical mapping data to visualise the core of re-entrant circuits at high levels of magnification.

The conduction block visualisation is inspired by the recently published Re-entry Vulnerability Index algorithm [7-9]. Using the index, the authors were able to highlight the vulnerability of different regions of tissue to initiation of re-entrant circuits by looking at the difference between activation and repolarisation times at pairs of points. Here, the algorithm has been adapted and expanded to account for the different features of the data collected at a monolayer level. The algorithm only uses activation times as repolarisation times are less likely to be robust in Ca^2+^ fluorescence optical mapping recordings due to the high affinity of Fluo-4 AM to Ca^2+^ [12]. Additionally, this method is performed on multiple successive time windows and the results combined, rather than processing a single time window as in the original work.

Briefly, the algorithm works as follows. An activation map is generated for one full rotation of the re-entry. For each point in the map ($i$), all nearby points ($j$) within a circular user-defined radius are found. Points are considered both ahead and behind the wavefront, rather than solely ahead as in the original Re-entry Vulnerability Index algorithm. For each of these pairs, the absolute activation time difference is calculated:

$$\mathrm{AT}_{\mathrm{diff}}=|\mathrm{AT}_{i}-\mathrm{AT}_{j}|$$

This value is added to a running total at the geometric midpoint between the two points. The process is repeated for each pixel in the activation map. Finally, the mean value at each point is calculated and can be plotted as a heatmap.

Figure 7a shows an activation time map for a re-entrant circuit. Figure 7b presents the heatmap which is the output of the above algorithm for one time window. The most obvious feature highlighted by this heatmap is the wavefront of propagation from the corresponding activation map. However, it also reveals part of the conduction block at the centre of the rotation.

**Fig. 7** (a) Activation map of re-entry circuit. (b) Heatmap of conduction block algorithm applied to one activation map. (c) Heatmap of conduction block algorithm applied to full recording of re-entry circuit

A novel aspect of this algorithm is isolation of the conduction block as the main feature in the final heatmap. The process described above is performed for all activation maps that can be generated in the recording. Time windows for each map are chosen for one full rotation of the circuit which can be determined by the dominant frequency of the re-entry. Finally, the mean value at each point through all maps is calculated and plotted. Figure 7c shows the final result of the algorithm applied to the full recording of a re-entry circuit. The line of conduction block in the re-entry can be clearly seen and ends with a small core of cells to the right of the map.

*Graphical User Interface*

The processes described above are combined into a graphical user interface to facilitate processing and analysis of high volumes of optical imaging data. The interface is designed and built in MATLAB using the GUIDE framework. Users can upload optical imaging recordings to complete the filtering processes and export analysis figures from within the program. Figure 8 shows the interface during processing of a re-entrant circuit recording. The software is available upon request.


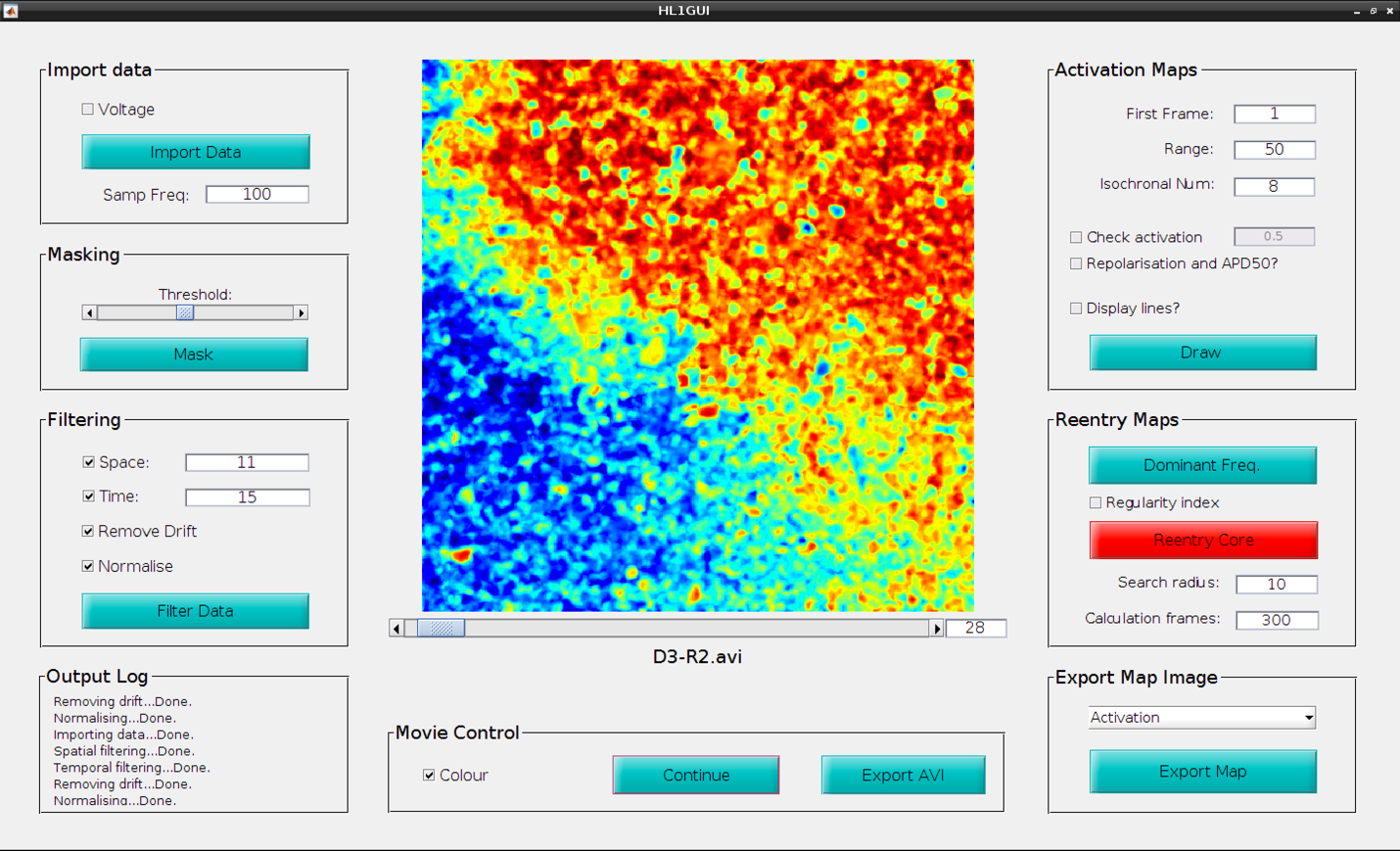


**Fig. 8** Image of graphical user interface developed to facilitate execution of optical mapping processing and analysis algorithms.

**References**

[1] J.I. Laughner, F.S. Ng, M.S. Sulkin, R.M. Arthur, I.R. Efimov, Processing and analysis of cardiac optical mapping data obtained with potentiometric dyes, Am. J. Physiol. Heart Circ. Physiol. 303 (2012) H753–65. doi:10.1152/ajpheart.00404.2012.

[2] C.H. Roney, Mathematical techniques for assessing cardiac wavefront dynamics, Bioengineering, 2015. http://hdl.handle.net/10044/1/39976.

[3] J. Canny, A computational approach to edge detection, IEEE Trans Pattern Anal Mach Intell. 8 (1986) 679–698.

[4] R. Telgársky, Dominant Frequency Extraction, arXiv. 1306 (2013) arXiv:1306.0103.

[5] J. Ng, J.J. Goldberger, Understanding and interpreting dominant frequency analysis of AF electrograms, J. Cardiovasc. Electrophysiol. 18 (2007) 680–685. doi:10.1111/j.1540-8167.2007.00832.x.

[6] P. Sanders, O. Berenfeld, M. Hocini, P. Jaïs, R. Vaidyanathan, L.-F. Hsu, et al., Spectral analysis identifies sites of high-frequency activity maintaining atrial fibrillation in humans, Circulation. 112 (2005) 789–797. doi:10.1161/CIRCULATIONAHA.104.517011.

[7] Y.R. Hill, N. Child, Ben Hanson, M. Wallman, R. Coronel, G. Plank, et al., Investigating a Novel Activation-Repolarisation Time Metric to Predict Localised Vulnerability to Reentry Using Computational Modelling, PLoS ONE. 11 (2016) e0149342–e0149342. doi:10.1371/journal.pone.0149342.

[8] N. Child, M.J. Bishop, B. Hanson, R. Coronel, T. Opthof, B.J. Boukens, et al., An activation-repolarization time metric to predict localized regions of high susceptibility to reentry, Heart Rhythm. 12 (2015) 1644–1653. doi:10.1016/j.hrthm.2015.04.013.

[9] R. Coronel, F.J.G. Wilms-Schopman, T. Opthof, M.J. Janse, Dispersion of repolarization and arrhythmogenesis, Heart Rhythm. 6 (2009) 537–543. doi:10.1016/j.hrthm.2009.01.013.

[10] P.V. Bayly, B.H. KenKnight, J.M. Rogers, R.E. Hillsley, R.E. Ideker, W.M. Smith, Estimation of Conduction Velocity Vector Fields from Epicardial Mapping Data, IEEE Trans. Biomed. Eng. 45 (1998) 563–571. doi: 10.1109/10.668746.

[11] F.J. Harris, On the use of windows for harmonic analysis with the discrete Fourier transform, Proc. IEEE. 66 (1978) 51–83. doi:10.1109/PROC.1978.10837.

[12] T.J. Herron, P. Lee, J. Jalife, Optical imaging of voltage and calcium in cardiac cells & tissues, Circulation Research. 110 (2012) 609–623. doi:10.1161/CIRCRESAHA.111.247494.
